# Systemic doxycycline as an adjunct to nonsurgical periodontal therapy in diabetic patients with periodontitis: a systematic review and meta-analysis

**DOI:** 10.3389/fphys.2024.1479152

**Published:** 2025-01-22

**Authors:** Zheng Zhang, Zhenyu Zhang, Guoquan Zhang

**Affiliations:** ^1^ Tianjin Stomatological Hospital, School of Medicine, Nankai University, Tianjin, China; ^2^ Tianjin Key Laboratory of Oral and Maxillofacial Function Reconstruction, Tianjin, China; ^3^ The School of Pharmacy, Jiamusi University, Jiamusi, China; ^4^ Jitang College, North China University of Science and Technology, Tangshan, China

**Keywords:** doxycycline, periodontitis, diabetes mellitus, meta-analysis, periodontal therapy

## Abstract

**Background:**

Recent studies that investigated the effects of systemic doxycycline as an adjuvant to scale and root planing (SRP) in the treatment of diabetic periodontitis have yielded controversial results. The aim of this meta-analysis is to evaluate the effect of systemic doxycycline as an adjunct to SRP against SRP alone for improving clinical outcomes of periodontitis in diabetic individuals.

**Methods:**

A systematic literature search was performed using PubMed, Cochrane library, China National Knowledge Infrastructure (CNKI), and VIP Data from the beginning of the database until March 2024. For probing depth (PD), clinical attachment level (CAL), plaque index (PI), gingival index (GI), and bleeding on probing (BOP), mean difference (MD) and the 95% confidence interval (CI) were computed. Heterogeneity was assessed using the Q test and the I^2^ statistic. Evaluation of publication bias was conducted using Egger’s and Begg’s tests.

**Results:**

A total of 12 articles were included for meta-analysis. No statistically significant difference was indicated in the improvement of PD, CAL, PI and GI between a treatment group receiving SRP combined with short-term antimicrobial dose doxycycline and controls receiving SRP alone. However, short-term antimicrobial dose doxycycline plus SRP significantly reduced BOP by 8.14% (95%CI 2.23–14.05) at 3 months. Furthermore, after the adjunctive use of long-term sub-antimicrobial dose doxycycline, significant reductions in GI (MD: 0.72, 95% CI: 0.34–1.10) and BOP (MD: 12.8, 95% CI: 0.24–25.36) were observed at 3 months. The robustness of the results was further confirmed by sensitivity analysis, despite the truth that significant heterogeneity was found among the included studies.

**Conclusion:**

Gingival inflammation in diabetic patients can be reduced more successfully by SRP combined with systemic doxycycline than by SRP alone, but this is insufficient in preventing periodontal tissue destruction.

## Introduction

Periodontitis is one of the most common chronic inflammatory diseases of humans, which is initiated by the microorganisms of the dental plaque (Zhang et al., 2020). If left untreated, it can harm the tooth-supporting structures and eventually lead to tooth loss (Al-Ahmari, 2021). Periodontitis is a major global public health challenge, with its severe types affecting 796 million people globally (Zhang et al., 2024). Systemic disorders have an impact on periodontitis, and diabetes mellitus is one of the underlying factors of this disease (Al-Ahmari, 2021). It is reported that periodontitis is more common and severe in diabetics than in nondiabetics (Alblowi et al., 2024). Patients with hyperglycemia have a greater risk of periodontal destruction due to a number of factors, including proinflammatory cytokine production, elevated cellular oxidative stress, and increased production and accumulation of advanced glycation end products in periodontal tissues (Javed et al., 2014).

Nonsurgical periodontal therapy (NSPT) is the cornerstone of periodontal therapy and the first recommended approach for the control of periodontal infections (Drisko, 2001). The efficacy of scaling and root planing (SRP) as a part of the NSPT of periodontitis has been established (Zhang et al., 2016). Although SRP improves clinical parameters, it may be inadequate, especially in particularly susceptible individuals, such as those with diabetes mellitus, to lower high levels of many underlying damaging inflammatory mediators (Alblowi et al., 2024). SRP with adjunct antibiotic therapy is usually performed, which helps in the extermination of pathogenic microbes from infected periodontal pockets (Alshehri and Javed, 2015). Some clinical trials have explored the use of antibiotics as an extra medication in NSPT for diabetic patients with severe periodontal disease (Tamashiro et al., 2016).

Doxycycline is an inexpensive, well-tolerated antibiotic that belongs to the tetracycline family and is effective against a wide range of bacteria, including those responsible for periodontal disease doses (Zaharescu et al., 2021). It has the added benefit of being a potent inhibitor of extracellular matrix, and can strongly inhibit the destruction of the matrix metalloproteinases (MMPs) (Alblowi et al., 2024). In the past decade, numerous clinical trials have been conducted to assess the efficacy of doxycycline used as an adjunct to SRP in the treatment of diabetic periodontitis. However, the sample size of these trials was small, the quality varied from low to high, and the results were inconsistent. We therefore aimed to examine the effect of systemic doxycycline when used as an adjunct to SRP on periodontal clinical data in periodontitis with diabetics by conducting a meta-analysis of randomized controlled clinical trials. Our hypothesis posits that the addition of systemic doxycycline to SRP will result in significantly improved periodontal clinical outcomes compared to SRP alone.

## Methods

This meta-analysis was carried out in accordance with the guidelines of Preferred Reporting Items for Systematic Reviews and Meta-Analyses (PRISMA). The final protocol was registered with PROSPERO (CRD42024626313).

### Search strategy

Two investigators (Z. Zhang and G. Zhang) independently conducted a comprehensive search of electronic databases, including PubMed, Cochrane library, China National Knowledge Infrastructure (CNKI) and VIP Data from their earliest records through 29 March 2024 for relevant studies. The search strategy was ((periodontitis) OR (periodontal disease) OR (Chronic Periodontitis) OR (Adult Periodontitis) OR (aggressive periodontitis) OR (Early-Onset Periodontitis) OR (Juvenile Periodontitis)) AND ((Doxycycline) OR (Vibramycin) OR (Atridox) OR (Doryx) OR (Hydramycin) OR (Oracea) OR (Periostat) OR (Vibravenos)). No language restrictions were applied. Furthermore, we also conducted manual searches.

### Study selection

The focused clinical question of this meta-analysis was as follows: “Does administration of a systemic doxycycline combination following NSPT in periodontitis individuals with diabetes lead to better clinical treatment outcomes compared to those who received NSPT alone?”

The inclusion criteria are based on the elements of the PICOS: Population (P), the study subjects were periodontitis patients with diabetes; Intervention (I), systemic doxycycline combination after NSPT; Comparison (C), NSPT alone, or in combination with placebo; Outcome (O), pocket probing depth (PD) and clinical attachment loss (CAL) were the primary outcome parameters, plaque index (PI), gingival index (GI), and bleeding on probing (BOP) were secondary outcome parameters; Studies (S), randomized controlled clinical trials. Studies were ruled out if they: 1) did not have a concurrent control group; 2) were not published as full reports; 3) or could not estimate outcomes based on the data presented.

### Data extraction

Data were collected and arranged in the following fields: first author, publication year, location, age, gender, patient number, intervention, periodontitis diagnosis, diabetes diagnosis, follow-up time and clinical outcomes. Two independent authors (Z. Zhang and G. Zhang) extracted relevant data from the included articles. Any discrepancy was resolved by discussion and consensus.

### Quality assessment

We used the Cochrane Collaboration’s tool for assessing risk of bias. The risk of bias assessment was undertaken by two of the authors (Z. Zhang and G. Zhang) independently. The assessment items include randomization, allocation concealment, blinding method, attrition bias, reporting bias and other bias. Method quality was evaluated as a percentage. Studies with percentages >50% were classified as high-quality studies, whereas studies with scores ≤50% were classified as low-quality studies.

### Statistical analysis

The changes in patients’ outcome parameters from baseline to final visit were compared between participants receiving doxycycline treatment (intervention group) vs. those receiving standard control therapies (control group). The results were reported as mean difference (MD) (intervention minus control) with 95% confidence interval (CI). Heterogeneity was tested by Q test and I^2^ analysis. A *p*-value more than 0.05 and I^2^ less than 50% was considered no statistically significant, the fixed effects model was used for analysis. Otherwise, the random effect model was performed, and further sensitivity analysis was used to find the source of heterogeneity. Publication bias was evaluated by Begg’s test and Egger’s test, and statistical significance was defined as a *p*-value <0.05. All analyses were performed using the STATA software (version 12.0, Stata Corp, College Station, TX, USA).

## Results

### Literature search

**FIGURE 1:**
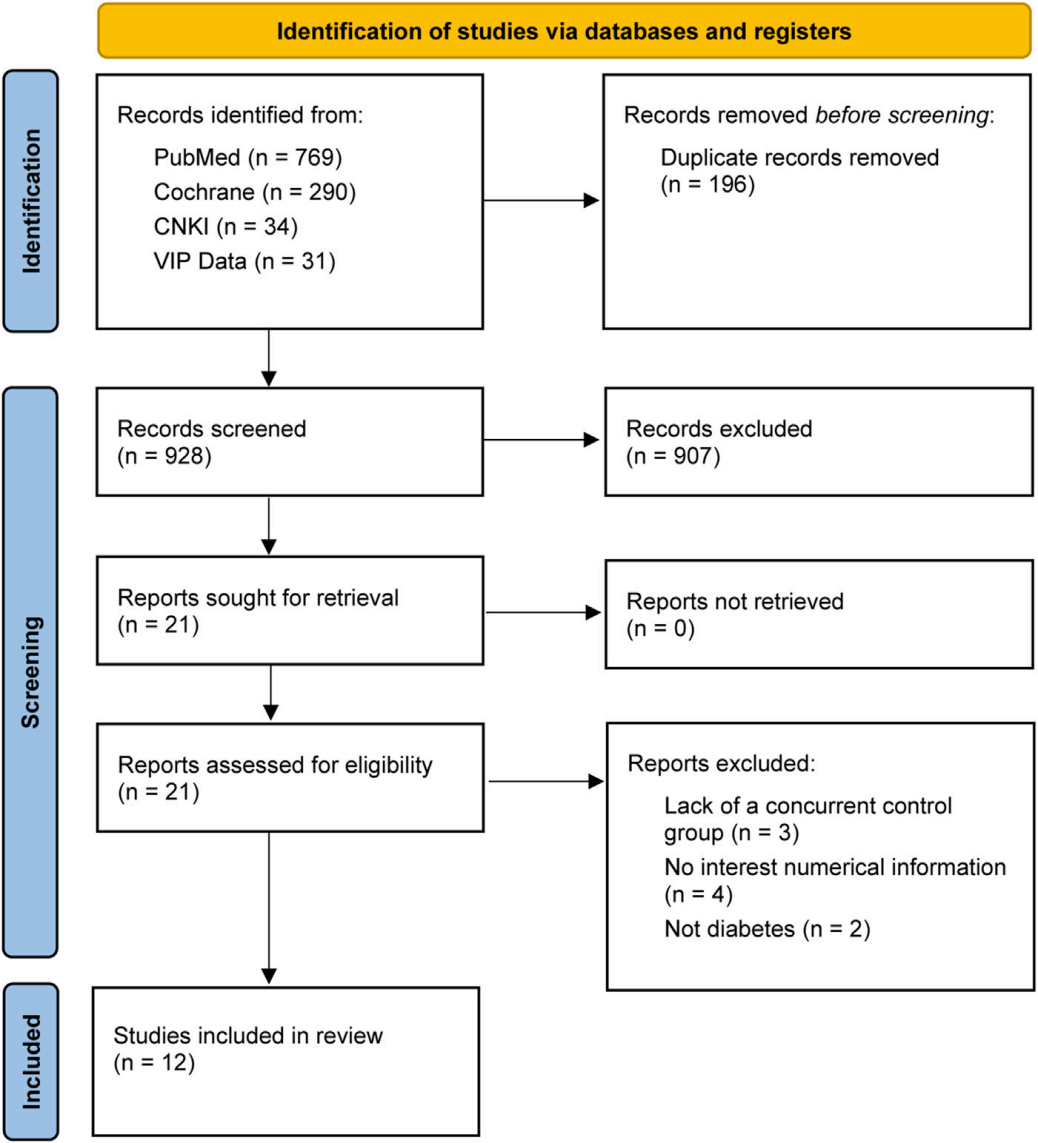
Flow chart from identification of eligible studies to final inclusion.

A total of 1,124 studies were retrieved from four electronic databases, and 21 full texts of these records were reviewed. Of these, nine full texts were not included in the final analysis for the following reasons: Absence of a concurrent control group (n = 3), lack of interest numerical information (n = 4), and failure to meet diabetes criteria (n = 2). Finally, 12 full texts were finally included in the meta-analysis (Figure 1).

### Characteristics of the included studies

A summary of the 12 included studies is given in Table 1. These studies were published between 2005 and 2024. All of the 12 studies were randomized controlled clinical trials. Four of the studies were conducted in Saudi Arabia (Al-Zahrani et al., 2009; Al-Nowaiser et al., 2014; Attia and Alblowi, 2020; Alblowi et al., 2024), four in India (Singh et al., 2008; Deo et al., 2010; Gaikwad et al., 2013; Das et al., 2019), and the remaining four trials were conducted in four different countries, including Spain (Llambés et al., 2005), Brazil (O'Connell et al., 2008), Poland (Gilowski et al., 2012) and Greece (Tsalikis et al., 2014). Ten trials were conducted in type 2 diabetes mellitus subjects with periodontitis, and one in diabetic type 1 patients with periodontitis. Eight trials involved short-term antimicrobial dose doxycycline (SADD), while four studies treated the subjects with long-term sub-antimicrobial dose doxycycline (LSDD).

**TABLE 1:** Characteristics of the studies included in the meta-analysis.

First author, (reference)	Year	Location	Age (years)	Female/Male	Control/Test no.	Medication regimen	Periodontitis type	Diabetes type	Follow-up time	Clinical outcomes
Fernando	2005	Spain	35.3	30/30	30/30	Control: SRP only Test: SRP + doxycycline 100 mg/day for 15 days after an initial dose of 200 mg	Moderate to severe	T1DM	3 months	PD, CAL, BOP
O’Connell	2008	Brazil	52.9	16/14	15/15	Control: SRP + Placebo Test: SRP + doxycycline 100 mg/day for 14 days after an initial dose of 200 mg	Moderate to severe	T2DM	3 months	PD, CAL, BOP
Singh	2008	India	>30	NA	15/15	Control: SRP only Test: SRP + doxycycline 100 mg/day for 14 days after an initial dose of 200 mg	Moderate to severe	T2DM	3 months	PI, GI, PD, CAL
Al-Zahrani	2009	Saudi Arabia	52.28	18/11	14/15	Control: SRP only Test: SRP + doxycycline 100 mg/day for 13 days after an initial dose of 200 mg	Moderate to severe	T2DM	3 months	PI, PD, CAL
Vikas	2010	India	37.1	12/8	10/10	Control: SRP + Placebo Test: SRP + doxycycline 20 mg twice daily for 6 months	NA	NA	6 months	PD, CAL
Gilowski	2012	Poland	36–68	18/16	17/17	Control: SRP + Placebo Test: SRP + doxycycline 20 mg twice daily for 3 months	PD ≥ 4 and CAL≥4	T2DM	3 months	PD, CAL, BOP
Gaikwad	2013	India	30–70	16/34	25/25	Control: SRP only Test: SRP + doxycycline 100 mg/day for 15 days	Generalized periodontitis	T2DM	1, 2, 3, 4 months	PI, GI, PD, CAL
Al-Nowaiser	2014	Saudi Arabia	42	26/42	35/33	Control: SRP only Test: SRP + doxycycline 100 mg/day for 14 days after an initial dose of 200 mg	Moderate to severe	T2DM	1, 3, 6 months	PI, GI, PD, CAL, BOP
Tsalikis	2014	Greece	>30	28/38	35/31	Control: SRP + Placebo Test: SRP + doxycycline 100 mg/day for 20 days after an initial dose of 200 mg	Moderate to severe	T2DM	3, 6 months	PD, CAL, BOP
Das	2019	India	≥30	NA	17/17	Control: SRP only Test: SRP + doxycycline 100 mg/day for 14 days after an initial dose of 200 mg	NA	T2DM	3 months	PI, GI, PD, CAL
Mai	2020	Saudi Arabia	36–48	13/17	15/15	Control: SRP + Placebo Test: SRP + doxycycline 20 mg twice daily for 3 months	Stage 2, grade B	T2DM	1, 3 months	PI, GI, PD
Jazia	2024	Saudi Arabia	25–55	9/6	5/5	Control: SRP + Placebo Test: SRP + doxycycline 20 mg twice daily for 3 months	Moderate to severe	T2DM	1, 3 months	GI, PD, CAL

BOP, bleeding on probing; CAL, clinical attachment loss; GI, gingival index; NA, not available; PD, probing depth; PI, plaque index; SRP, scaling and root planning; T1DM, type 1 diabetes mellitus; T2DM, type 2 diabetes mellitus.

### Quality assessment of the included studies

**FIGURE 2:**
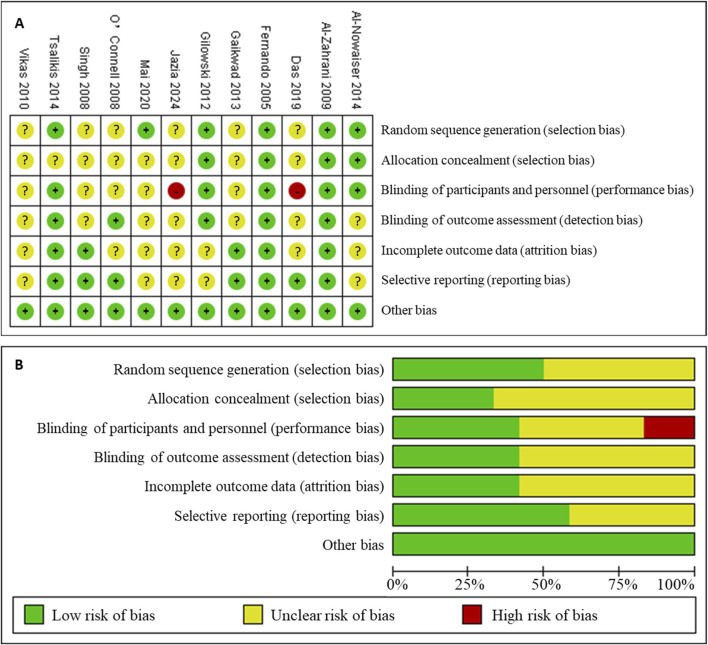
Risk of bias assessment for the studies included in the meta-analysis. **(A)**, risk of bias summary; **(B)**, risk of bias graph. (+): low risk of bias; (?): unclear risk of bias; (−): high risk of bias.

The risk of bias in the included studies is summarized in Figure 2A and an overall assessment of risk of bias is presented in Figure 2B. Out of the 12 articles, six had clear method of random sequence. Four articles described the procedure to achieve allocation concealment. Five studies reported personnel, participants and assessor blinded to treatment. More than 50% of the articles were regarded as low risk of bias in reporting bias.

### Meta-analysis of primary outcome

**FIGURE 3:**
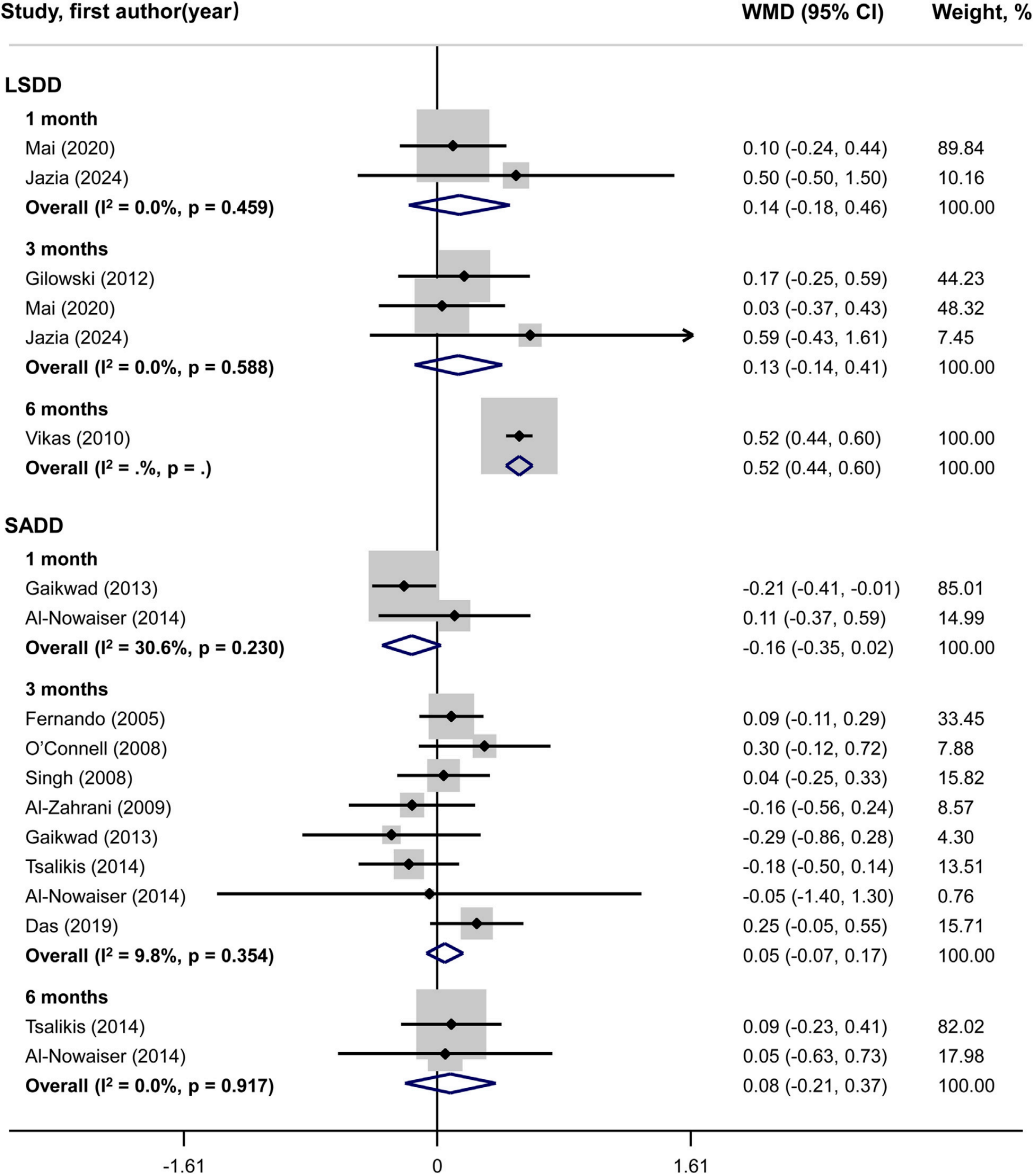
Forest plot of probing depth (PD). LSDD, Long-term sub-antimicrobial dose doxycycline; SADD, Short-term antimicrobial dose doxycycline; WMD, Weighted mean difference.

**FIGURE 4:**
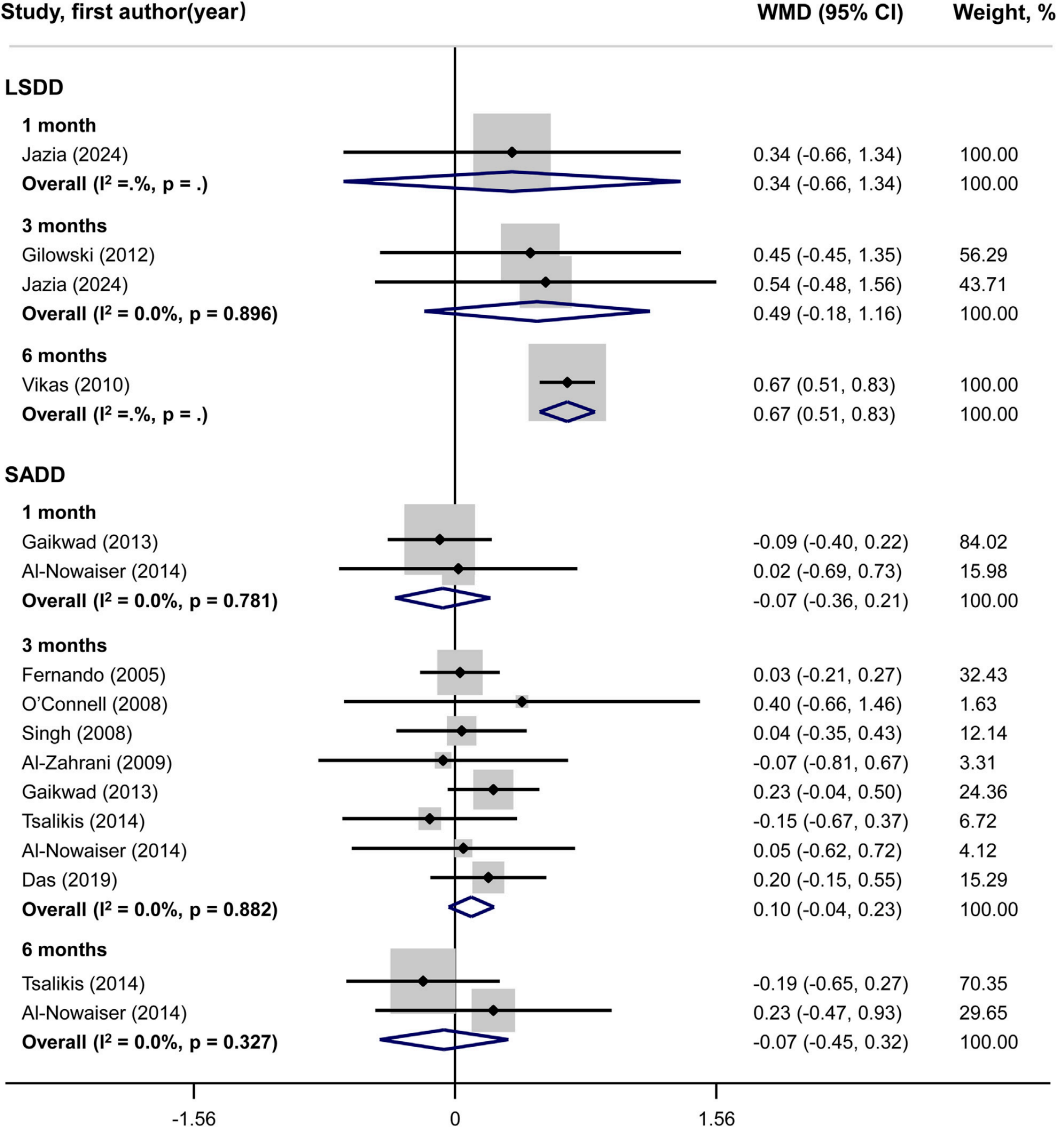
Forest plot of clinical attachment level (CAL). LSDD, Long-term sub-antimicrobial dose doxycycline; SADD, Short-term antimicrobial dose doxycycline; WMD, Weighted mean difference.

Systemic doxycycline as an adjunct to SRP did not yield significant differences in PD and CAL outcomes compared to SRP alone, according to SADD studies. In LSDD studies, however, a statistically significant difference in PD (WMD = 0.52; 95%CI: 0.44, 0.60) and CAL (WMD = 0.67; 95%CI: 0.51, 0.83) changes was observed across groups only at 6 months, but not at 1 month or 3 months. Neither the LSDD nor the SADD studies showed statistically significant heterogeneity among the studies regarding PD and CAL outcomes, with all P-values exceeding 0.05 and I^2^ less than 50% (Figures 3, 4).

### Meta-analysis of secondary outcomes

**FIGURE 5:**
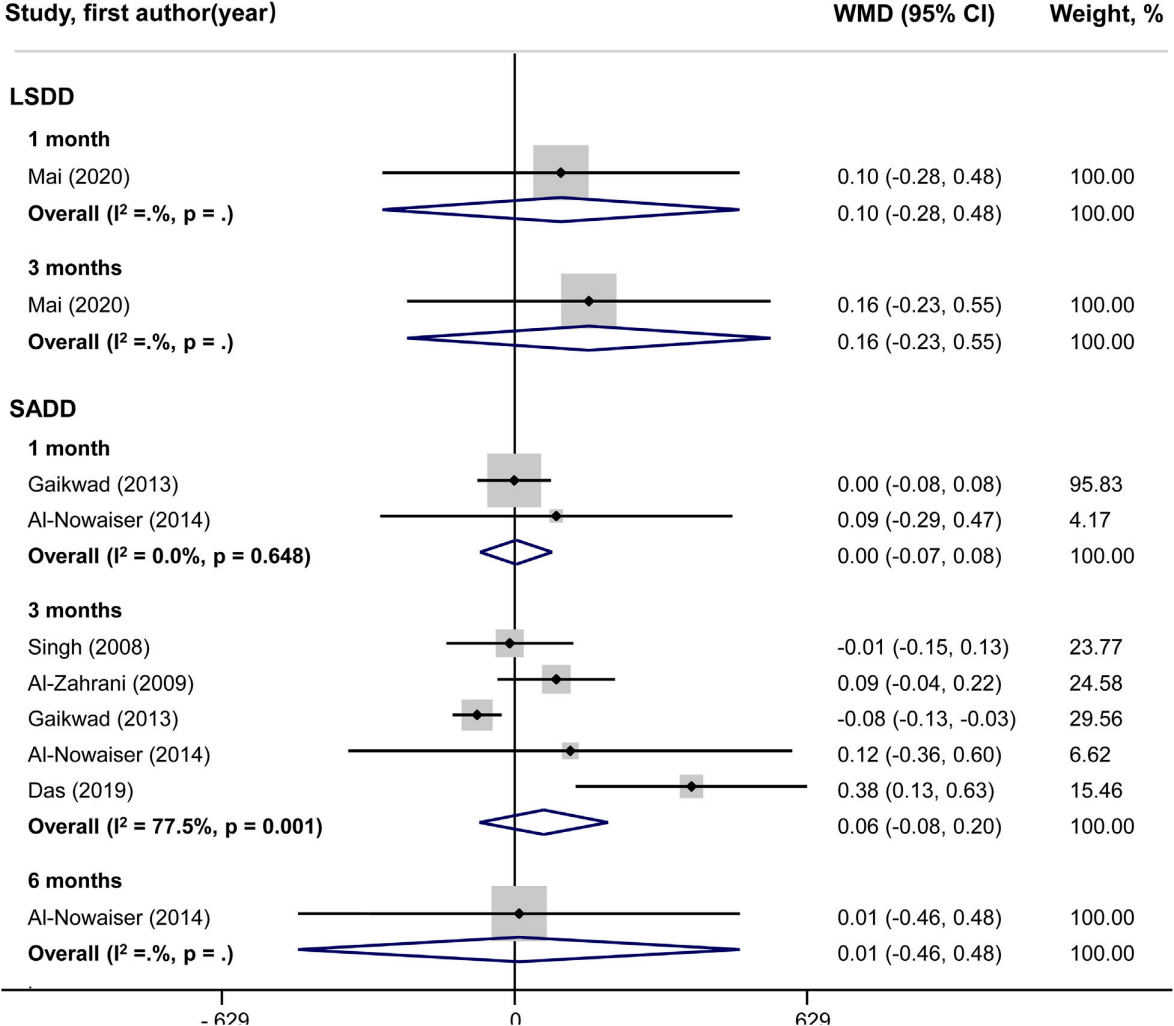
Forest plot of plaque index (PI). LSDD, Long-term sub-antimicrobial dose doxycycline; SADD, Short-term antimicrobial dose doxycycline; WMD, Weighted mean difference.

**FIGURE 6:**
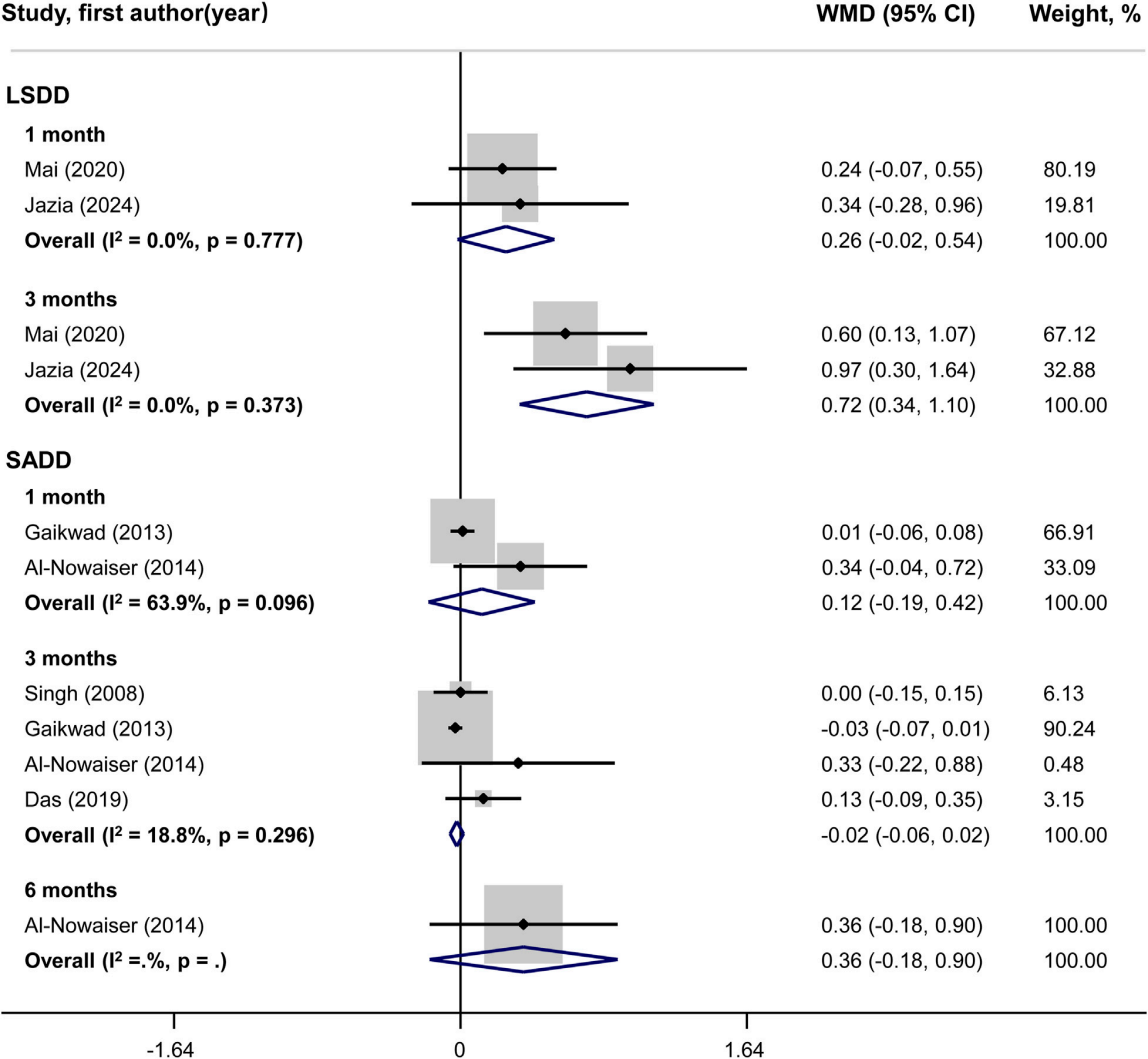
Forest plot of gingival index (GI). LSDD, Long-term sub-antimicrobial dose doxycycline; SADD, Short-term antimicrobial dose doxycycline; WMD, Weighted mean difference.

**FIGURE 7:**
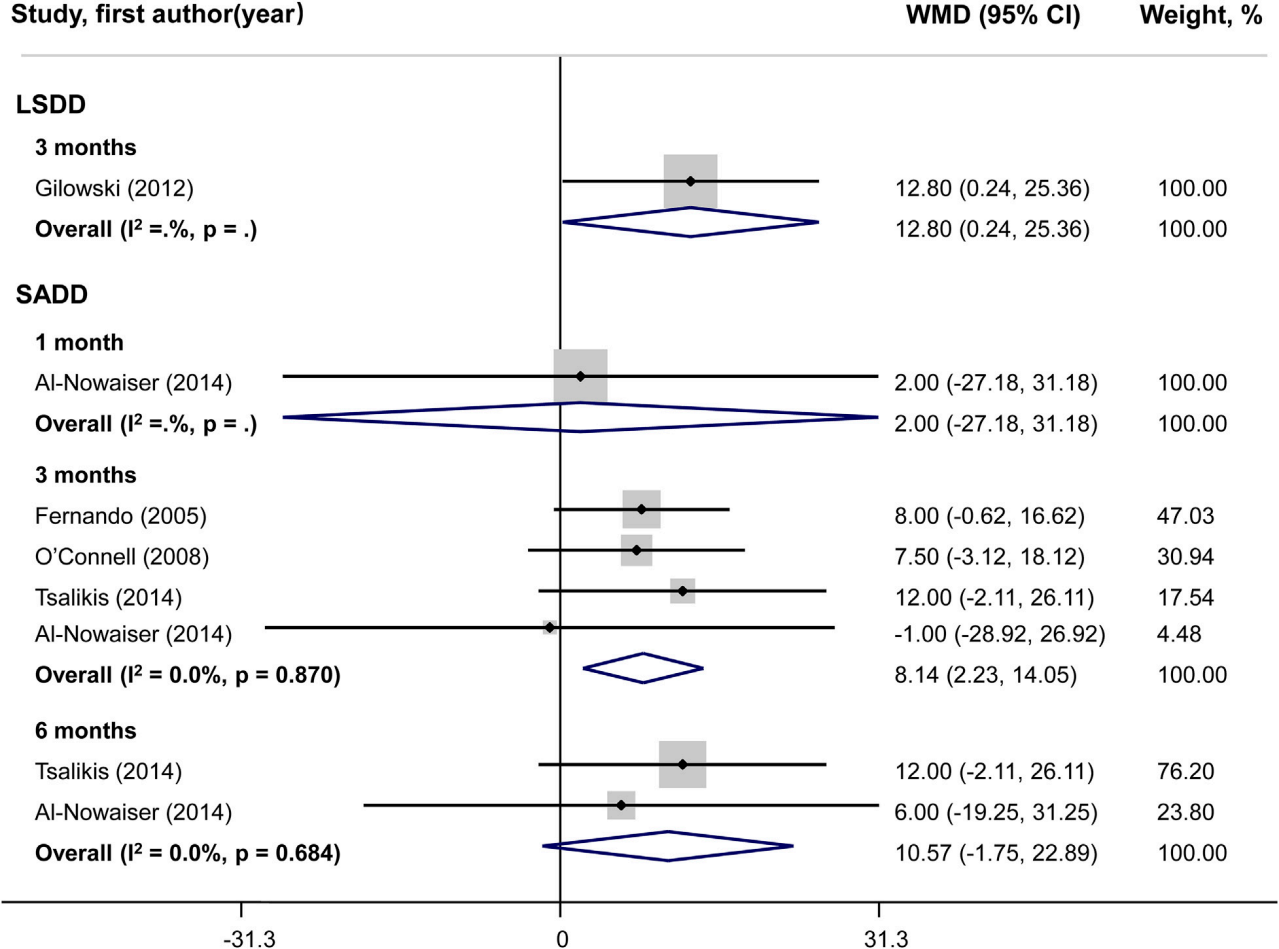
Forest plot of bleeding on probing (BOP). LSDD, Long-term sub-antimicrobial dose doxycycline; SADD, Short-term antimicrobial dose doxycycline; WMD, Weighted mean difference.

No statistically significant difference could be shown for PI changes between groups in both LSDD and SADD studies (Figure 5). Systemically administered doxycycline was statistically significantly associated with GI reduction (WMD = 0.72; 95%CI: 0.34, 1.10) in 3-month LSDD experiments but not in SADD investigations (Figure 6). Besides, both the LSDD and SADD studies showed statistically significant improvement in BOP when systemic doxycycline is used as an adjunct to SRP compared to just SRP alone at 3 months (Figure 7). No statistically significant heterogeneity could be shown for PI, GI and BOP in LSDD studies (I^2^ < 50% and *P* > 0.05). But SADD studies revealed significant heterogeneity for PI (I^2^ = 77.5%, *P* = 0.001) at 3 months and GI (I^2^ = 63.9%, *P* = 0.096) at 1 month.

### Sensitivity analysis

**FIGURE 8:**
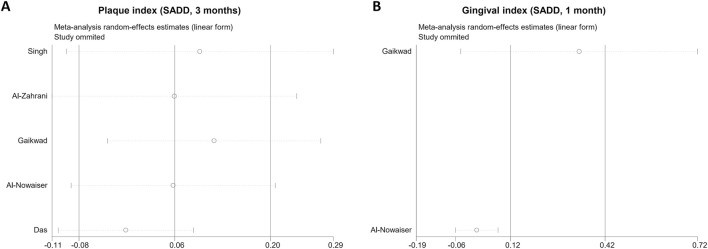
Sensitivity analysis of clinical indices. **(A)**, plaque index; **(B)**, gingival index. SADD, Short-term antimicrobial dose doxycycline.

In SADD studies, heterogeneity appeared significant for PI at 3 months and GI at 1 month, so we conducted further sensitivity analysis. The results of the sensitivity analysis showed that PI and GI results were stable, and excluding any single study did not have a significant impact on the experimental results (Figures 8A, B).

### Publication bias

Two tests, Begg’s and Egger’s, were employed to evaluate publication bias. Neither test detected any evidence of publication bias in the changes observed for PD, CAL, PI, GI, and BOP. All P-values were greater than 0.05 (Table 2).

**TABLE 2:** Publication bias in meta-analysis.

Publication bias (P-value)	Begg’s test			Egger’s test		
	1 month	3 months	6 months	1 month	3 months	6 months
LSDD						
Plaque index	NA	NA	NA	NA	NA	NA
Gingival index	1.00	1.00	NA	NA	NA	NA
Probing depth	1.00	0.30	NA	NA	0.27	NA
Attachment loss	NA	1.00	NA	NA	NA	NA
Bleeding on probing	NA	NA	NA	NA	NA	NA
SADD						
Plaque index	1.00	0.46	NA	NA	0.10	NA
Gingival index	1.00	0.31	NA	NA	0.07	NA
Probing depth	1.00	0.54	1.00	NA	0.49	NA
Attachment loss	1.00	0.90	1.00	NA	0.76	NA
Bleeding on probing	NA	0.73	1.00	NA	0.57	NA

LSDD, Long-term sub-antimicrobial dose doxycycline; NA, not available; SADD, Short-term antimicrobial dose doxycycline.

## Discussion

In the literature, data regarding the efficacy of systemic doxycycline as an adjunct to SRP in the treatment of diabetic periodontitis are contradictory. Some of the studies revealed a marked improvement in periodontal clinical data following the use of adjunctive doxycycline in SRP (Deo et al., 2010; Gilowski et al., 2012; Das et al., 2019), but others failed to find any evidence of improvement (Tsalikis et al., 2014; Attia and Alblowi, 2020). Therefore, it is necessary to do this meta-analysis, which identified 12 randomized controlled studies evaluating the efficacy of doxycycline as an adjunct to SRP. The findings from the present meta-analysis showed that SADD plus SRP, compared with SRP alone, did not produce a significant improvement in PD, CAL, PI and GI, but an additional 8.14% (95%CI 2.23–14.05) of BOP reduction at 3 months.

CAL is the gold standard for diagnosis of periodontitis, PD is also as important as CAL since deep pockets raise the possibility of developing periodontal disease, which indicates a need for additional treatment (Matuliene et al., 2008; Matuliene et al., 2010). At the same time, PD reduction is considered as an important aspect for evaluation of the success of periodontal therapy (Das et al., 2019). Therefore, CAL and PD were identified as the primary outcome parameters for the present meta-analysis. However, our investigation showed no significant improvements in either PD or CAL in diabetic patients with periodontitis who are receiving SADD as an adjunct to SRP. The current evidence suggests that SADD may not offer significant benefits in terms of PD and CAL improvements in diabetic patients with periodontitis.

LSDD is an already approved medication for the adjunctive treatment of periodontitis (Engebretson and Hey-Hadavi, 2011). In the last few decades, many clinical studies have described the beneficial effects of LSDD adjunctive to SRP on the basis of the significant reduction in periodontal indices that is observed with this treatment (Pârvu et al., 2013). Moreover, a previous meta-analysis of multiple studies has demonstrated that LSDD treatment combined with NSPT could significantly improve clinical parameters, reduce PD, and gain CAL in periodontitis patients without diabetes (Sgolastra et al., 2011). In contrast, our meta-analysis focused on diabetic patients. Although LSDD as an adjuvant to SRP was found to significantly improve PD and CAL after 6 months of follow-up in the treatment of periodontitis, the results were insufficient since only one study was included in the data analysis. Future studies with larger sample sizes and longer follow-up periods are necessary to further explore the efficacy of these interventions.

Previous study has shown individuals with type 2 diabetes who receive LSDD treatment in addition to NSPT exhibit decreased HbA1c levels; however, no such reduction in HbA1c levels was observed in those who receive SADD at that time (Engebretson and Hey-Hadavi, 2011). The choice of medication regimen (LSDD or SADD) may significantly influence the outcomes of HbA1c changes. Comparable findings were observed in the analysis of the GI, an indicator of gingival inflammation. In our meta-analysis, we found that the combination of LSDD treatment with SRP had a beneficial effect on reducing GI at 3 months. In contrast, the use of SADD in combination with SRP did not demonstrate a statistically significant improvement in GI compared to SRP alone in diabetic patients. The implications of these findings suggest that systemic doxycycline, when applied through the LSDD method, may play a pivotal role in managing periodontal health among individuals with diabetes.

Besides GI, BOP is also the most frequently utilized indicator for the existence of an inflammatory lesion in the gingival tissue (Thoma et al., 2018). As shown in previous meta-analyses, there was no statistically significant difference in the improvement of BOP with the use of systemic doxycycline as an adjunct to SRP (Yap and Pulikkotil, 2019). However, the evidence of the meta-analysis mentioned above needs to be strengthened, as only two studies were used for the quantitative synthesis of BOP (Yap and Pulikkotil, 2019). In our meta-analysis, five studies were included in BOP quantitative synthesis, and the adjunct of systemic doxycycline (both LSDD and SADD) to SRP was found to have an additional benefit in reducing BOP level at 3 months. The results of our investigation indicate that NSPT in combination with systemic doxycycline could alleviate gingival inflammation in diabetes patients.

In the course of the present investigation, several limitations must be recognized. To begin with, the criteria employed for diagnosing periodontitis varied considerably. This inconsistency in diagnostic criteria may have introduced a degree of bias into the study, as different standards for identifying periodontitis could lead to differences in patient selection and treatment outcomes. Another noteworthy limitation is the language bias in our meta-analysis. The vast majority of the studies included in our review were published in English. This may have excluded valuable research conducted in other languages, potentially limiting the comprehensiveness of our findings. Additionally, the robustness of the results of some comparisons could be affected by the heterogeneity of the studies. This heterogeneity introduces variability that may obscure the true effects being investigated. Finally, the relatively small number of studies included in the analysis led to an insufficient sample size, which in turn limits the statistical power of the study.

## Conclusion

This meta-analysis of randomized controlled clinical trials provides evidence that when combined with systemic doxycycline, SRP is more effective than when used alone to alleviate gingival inflammation in diabetic individuals; yet it is not sufficient to significantly prevent the destruction of periodontal tissue. Additional long-term studies with larger sample sizes should be conducted to confirm this statement.

Clinical Relevance: When used in conjunction with SRP, systemic doxycycline did not considerably prevent the progression of periodontitis in diabetic patients.

## Data Availability

The original contributions presented in the study are included in the article/supplementary material, further inquiries can be directed to the corresponding authors.
